# Rapid eye movements to a virtual target are biased by illusory context in the Poggendorff figure

**DOI:** 10.1007/s00221-015-4263-3

**Published:** 2015-04-26

**Authors:** D. Melmoth, S. Grant, J. A. Solomon, M. J. Morgan

**Affiliations:** Division of Optometry and Visual Science, City University London, London, UK; Max-Planck Institute for Metabolism Research, Cologne, Germany

**Keywords:** Perception, Action, Saccades, Poggendorff illusion

## Abstract

In order to determine the influence of perceptual input upon oculomotor responses, we examined rapid saccadic eye movements made by healthy human observers to a virtual target defined by the extrapolated intersection of a pointer with a distant landing line. While corresponding perceptual judgments showed no evidence of systematic bias, eye movements showed a strong bias, in the direction of assimilation of the saccade trajectory to the shortest path between the end of the pointer and the landing line. Adding an abutting vertical inducing line to make an angle of 45 deg with the pointer led to a larger bias in the same direction as the classical Poggendorff illusion. This additional Poggendorff effect was similar in direction and magnitude for the eye movements and the perceptual responses. Latency and dynamics of the eye movements were closely similar to those recorded for a control task in which observers made a saccade from the start fixation to an explicit target on the landing line. Further experiments with inducing lines presented briefly at various times during the saccade latency period showed that the magnitude of the saccade bias was affected by inducer presentation during the saccade planning process, but not during the saccade itself. We conclude that the neural mechanisms for extrapolation can feed into the control of eye movements without obvious penalties in timing and accuracy and that this information can instantaneously modify motor response throughout the planning phase, suggesting close association between perceptual and motor mechanisms in the process of visuo-spatial extrapolation.

## Introduction

The original version of the two-stream hypothesis of visual cortical function (Goodale et al. 1994; Goodale and Milner 1992; Milner and Gooddale 1995) postulated that the dorsal and ventral streams independently process visual information for action and perception. Despite some evidence in support of this segregation from the effect of visual illusions upon perception, but not upon action (Aglioti et al. 1995; Haffenden and Goodale 1998; Haffenden et al. 2001; Mack et al. 1985; Servos, 2000; Westwood et al. 2000a, b), there is now ample evidence that under certain conditions illusions can affect both perception and action equally, suggestive of similar visual representations for perception and action (Daprati and Gentilucci 1997; de Grave et al. 2004; Franz 2003a, b; Franz et al. 2000, 2003; Gentilucci et al. 1996; Melmoth et al. 2009; Predebon 2004; Smeets et al. 2002; van Donkelaar 1999). The conditions under which perceptual biases are, or are not, accompanied by motor effects thus require further investigation.

**Fig. 1:**
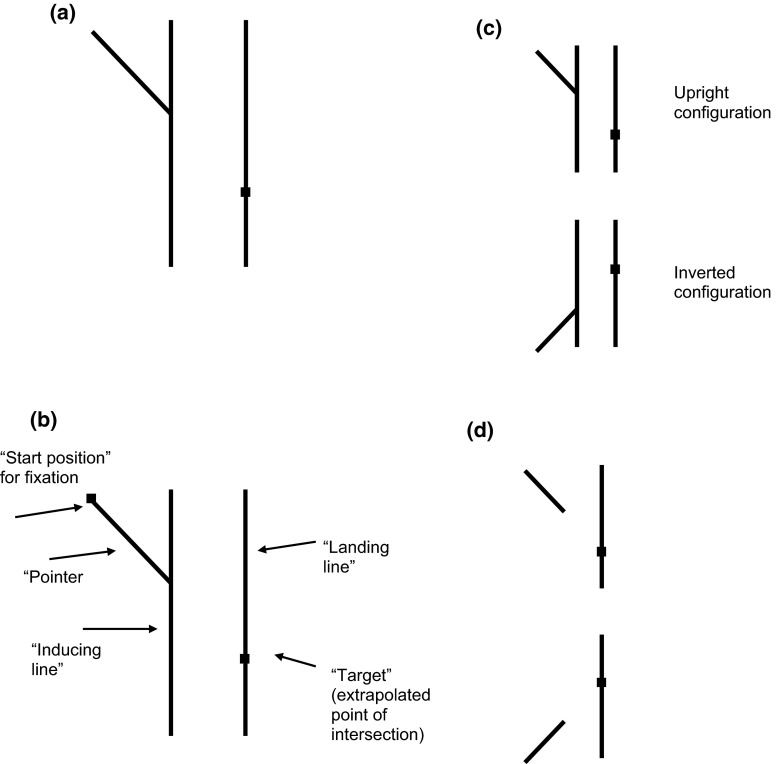
Stimulus configuration. **a** The Poggendorff illusion, with the inducing line at 45° to the pointer. Typically, participants perceive the correct extrapolated intersection on the landing line (shown by the *black square*) as being too low. **b** The stimulus components. **c** Stimuli were presented in both the upright and inverted configuration; **d** the ‘pointer-only’ condition

Melmoth et al. (2009) measured rapid manual pointing of normal healthy participants in a visuo-motor extrapolation task (Fig. 1). Specifically, the target was defined by the virtual intersection of a 45° oblique ‘pointer’ line and a distant vertical ‘landing’ line. The ability of the participants to perform this extrapolation task was also measured by a perceptual response, in which they had to adjust the position of a marker placed on the landing line to their estimated position of its virtual intersection with the distant pointer. In the presence of the pointer and landing line alone (Fig. 1d), perceptual judgments were near veridical, while motor responses showed an extrapolation bias, which took the direction of underestimating the length of the vector joining the tip of the pointer to the landing line, or in other words, assimilating the vector to the shortest distance between pointer and landing line. When a vertical inducing line at 45° to the pointer was added (Fig. 1a, b), the bias for both motor and perceptual responses increased, but by similar magnitudes in each case. Thus, the total motor bias was once again greater than the perceptual bias. This suggested that two independent additive biases were at work: an extrapolation bias in the presence of the pointer and landing line alone (which affected only motor responses) and the classical Poggendorff bias caused by the inducing line, which affected both response modes equally. Melmoth et al. conjectured that there was a unique motor bias which took form of a principle of least effort, in agreement with many studies of movement ‘undershooting’ (e.g., Harris 1995) but that otherwise, the Poggendorff effect was very similar to motor and perceptual responses, suggestive of a common neural mechanism. The dynamics of the pointing response to the virtual target, including the latency and peak velocity, were indistinguishable from those to an explicit target, arguing against different mechanisms for the two kinds of pointing.

In the present study, we extended the extrapolation method to saccadic eye movements. We wished to know how quickly and accurately human subjects could make a saccade along a path defined by a pointing line and terminate the saccade on a landing line. We also wished to determine whether the saccade would be influenced by an inducing context. An important difference between the saccade task and the manual pointing used by Melmoth et al. (2009) is that the saccade task is open loop, whereas in the manual pointing task subjects could see their hand moving to touch the stimulus configuration so this involved an element of closed-loop (feedback) control. There is some evidence that the open-loop conditions of saccade control may favor effects of contextual illusions on performance (de Grave et al. 2006a, b; Knox and Bruno 2007) while, conversely, it is known that several illusions are reduced by active saccade-driven visual exploration (Gillam 1980; Gillam and Chambers 1985; Tibber et al. 2009). We therefore had participants make a saccade from a start position at the end of an oblique pointer to its projected intersection with a distant vertical landing line as soon as these components appeared on the screen. The movement was made either to an explicitly marked point on the landing line or to a virtual target which the participant had to compute by visual extrapolation from the pointer to the landing line, with the extra inducing line absent or present on different trials.

An important aim of the study was the compare the characteristics of saccades to an explicit and a virtual target, especially the saccade latency. If an extra perceptual process is involved in planning a saccade to a virtual target, we predicted that the latency of saccades to a virtual (extrapolation) target should be greater than those to an explicit dot target.

## Methods

### Participants

In experiment 1, there were five participants. Two were the authors MM and DM; the others were postdoctoral vision researchers naïve to the specific aims of the study. The same authors and two of the naïve subjects also participated in experiment 2. All participants had normal or corrected-to-normal vision. Informed consent was obtained prior to inclusion, and procedures were in accordance with the Declaration of Helsinki.

### Stimuli and display

Stimuli were presented on a vertically oriented Protouch 17-inch TFT flat-screen display, via a PC fitted with a VSG graphics card (Cambridge Research Systems Ltd., Rochester, UK) running custom-written scripts for MATLAB (MathWorks Ltd., Cambridge, UK). On-screen pixel size was 0.36 mm, and average background luminance was 55 cd/m^2^, while average luminance of the stimulus components was 130 cd/m^2^. The inducing and landing lines were vertical and measured 25.4 cm × 0.07 cm with a 7.3-cm separation in the parallel conditions. The oblique pointer was 5.1 cm long (approx. 5.9°) and angled at −45° or +45° relative to the horizontal for top-down (upright) or bottom-up (inverted) configurations (Fig. 1c), respectively. Next to the landing line centered 50 pixels to the right was a numeral scale (1–5) which participants used to report for their perceptual response. Numeral height was 7 mm. Participants were loosely restrained with a chin rest and viewed the display at a distance of 50 cm so that the stimulus dimensions in degrees of visual angle were approximately 28.5°, 0.08° and 8.4°, respectively, with numeral height of approximately 0.8°. There were four main stimulus conditions in total, with five trials per condition randomly interleaved. On half of the trials, the start position was the bottom-left quadrant of the screen and the saccade moved upward and to the right. On the other half of the trials, the start position was in the top-left quadrant of the screen and the saccade was moved downward and to the right. Since the expected direction of the Poggendorff bias is expected to reverse between the two directions, we took a measure of mean bias consisting of (upward–downward)/2. On half of the trials, the inducing line was present; on the other half, it was absent. Finally, in additional probe trials, there was an explicit target for the saccade, equal in size to the fixation point, and placed on the landing line at the veridical point of extrapolation of the pointer, which was presented without an inducing line. The on-screen position of the entire stimulus configuration was spatially jittered from trial to trial, in order to minimize stereotypical movements and practice effects.

### Eye movement recording

Point of regard (POR) was measured by an infrared, corneal reflex video-camera system (IScan RK-464, Iscan Inc., Burlington, Ind. USA) at a frame rate of 50 Hz. Because we were interested in the absolute spatial accuracy of eye position rather than in the shape of the saccade trajectory, it was essential to calibrate the POR in display screen coordinates. Each session began with a calibration in which the observer fixated in succession at a central fixation point and four points arranged in a square (18.5° × 18.5°). Because the [x y] coordinates measured in this way seldom corresponded exactly with the relative screen positions, they were linearly transformed by multiplication with a 2 × 2 matrix to form a square array with the fixation point in the middle. The best-fitting matrix was found by the MATLAB ‘fminsearch’ procedure. The efficacy of the transformation was then tested by having the observer to fixate some other arbitrary point inside the square. If the transformed position of the POR relative to the center of the square differed by <5 % (Euclidean distance) from the actual position, the experiment was started, and otherwise, the calibration was repeated. Furthermore, at the start of each trial, the observer was instructed to fixate a bright spot on the distal end of the pointer. If transformed POR differed by more than 5 % from its actual position (which was jittered from trial to trial), a new calibration was undertaken. This happened on average about once every session, but it was not unusual to have whole sessions in which a single calibration was maintained throughout.

The criterion for the end of the first saccade was that velocity fell below 30°/s for a period of 20 ms. No further saccades were included in the analysis.

### Procedure

At the beginning of each trial, a small 8 × 8 pixel fixation point appeared at either the top-left or bottom-left of the screen (50 % each, randomly presented). Once the real-time POR measurement confirmed that participants were fixating on the correct position, the stimulus appeared and this was the participants’ cue to begin their saccade. The stimulus consisted of the oblique (45°) pointer with its left-hand end aligned with fixation (see Fig. 1), the landing line and occasionally an explicit marker which was present on 20 % of trials to establish baseline accuracy and to allow comparison between saccades made to implicit versus explicit target locations. Presentation of the abutting inducing line differed between experiments. For experiment 1, the inducer was present for the entire duration of the trial (900 ms). For experiment 2, there was an onset delay for the inducer following the appearance of the rest of the stimulus. All stimulus components were extinguished after 900 ms. Once participants had completed their eye movement, they were required to report a verbal estimate of the position of the intersection, relative to the numerical scale to the right of the landing line, giving a number between 0 and 5, to one decimal place. In all experiments, control trials measured responses in the absence of any inducing line, i.e., pointer only.

## Experiment 1

### Results

**Fig. 2:**
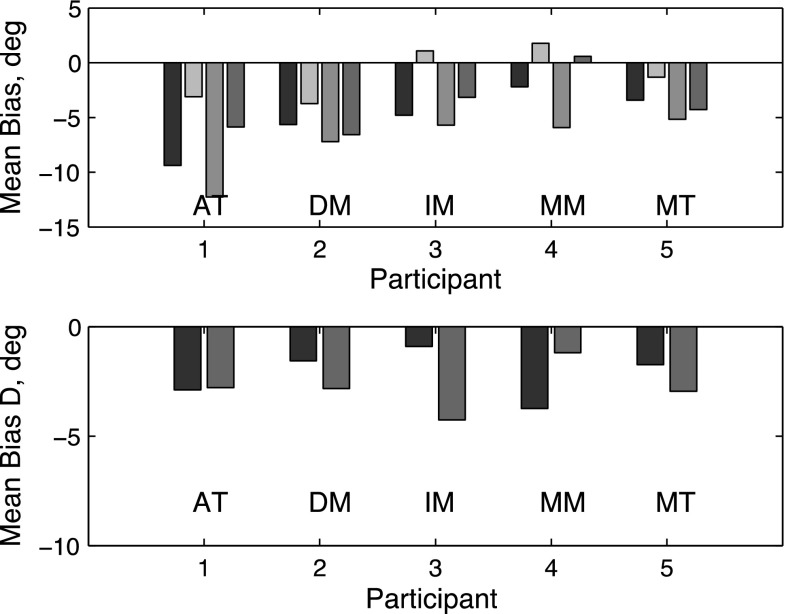
*Top panel* of the figure shows the mean bias in experiment 1, combining results from the upward and downward conditions (u–d)/2. The *colored bars* (reading *left* to *right*) show results for pointer-only, motor (*dark blue*), pointer-only perceptual (*light blue*), with inducer, motor (*yellow*) and with inducer, perceptual (*brown*) conditions. Results are shown separately for each participant. *Negative values* on the vertical axis represent a shift in the direction of the classical Poggendorff effect. The *bottom panel* shows the difference (D) between the with-inducer and no-inducer conditions for motor (*dark blue*) and perceptual (*brown*) responses, in order to isolate the Poggendorff bias from the undershoot effect. Note the difference in *y*-axis scale between *top* and *bottom panels* (color figure online)

We define the Poggendorff bias in the saccade as the difference in angle (deg) between the true vector from the start position to its extrapolated intersection with the landing line and the vector joining the start position to the point of regard upon the landing line following the initial saccade. The perceptual Poggendorff bias is defined similarly, using the observer’s report of the apparent point of alignment. The results for individual participants (Fig. 2) showed strong saccade direction biases in the Poggendorff direction in both the inducer-absent and inducer-present conditions. The perceptual effect was smaller than the saccade effect in all cases.

These results are in good agreement with our previous findings for manual pointing (Melmoth et al. 2009). Two-factor ANOVA confirmed significant main effects of both response modes (perceptual versus motor *F*[1,4] = 13.4, *p* = 0.021), and the presence or absence of the inducing line (*F*[1,4] = 456.1, *p* < 0.0001), and with no interaction between the two factors. Subtracting each participant’s baseline (i.e., pointer-only) bias reveals the amount of additional bias attributable solely to the Poggendorff inducing line. This averaged 4.4° versus 5.7° for motor and perceptual response modes, respectively, the small difference between them being non-significant.

These data support the conjecture that there are two independent biases at work: one depending on the oblique pointer (and primarily affecting the motor response) and the other depending on the inducing line (affecting both response modes approximately equally). When the inducing line is absent, we see only the effect of the oblique pointer. When the inducer is present, we have an additional Poggendorff effect of the inducing line.

One participant MM showed only a small perceptual Poggendorff effect (~1°). This may have been due to the novel method of measuring the effect, which consisted of making an alignment decision *after* making a saccade to the landing line, using a numerical scale not on the line itself. Another explanation is that this observer has had extensive practice with the Poggendorff alignment task (Morgan 1999; Morgan et al. 2013), and practice is known to reduce the strength of a variety of geometrical illusions (Judd 1902; van der Kamp et al. 2013). The size of effect reported for this same subject (identified as ‘MJM’) using the ‘comparisons of comparisons’ method (Morgan et al. 2013) was 3.4°; more recent measurements (unpublished) using the same method have given values of 1° or less. If the diminution of the perceptual effect is really due to practice, it is of potential interest that the size of the motor effect for MM in the present experiment was similar to that of the other four participants.

### Explicit versus implicit target conditions and eye movement characteristics

There were no systematic biases in the condition where the saccade was made to an explicit target point on the landing line. Key characteristics of the eye movements were measured to determine whether they were affected by the differing target conditions: latency (time to movement onset in msec); peak velocity (degrees of visual angle per second), time taken to reach the point of maximum velocity (ms) and finally the time spent after maximum velocity in the deceleration phase up to the end of the saccade (ms). Table 1 shows that these measures for saccades to implicit (extrapolated) compared to explicit targets were similar, with paired *t* tests revealing no significant differences between them.

**Table 1:** Mean values across trials for saccades to implicit targets (where the participant had to extrapolate the intersection between the oblique pointer and the landing line) and explicit targets which were indicated by a marker on the landing line

Participant	Latency (ms)	Peak vel (°/s)	Time to PV (ms)	Post PV (ms)
Implicit target				
1	285	138	313	183
2	337	161	368	148
3	322	236	178	228
4	300	160	208	235
5	253	181	167	208
Mean	299	175	247	201
Explicit target				
1	282	152	262	218
2	347	160	312	173
3	338	205	163	223
4	287	148	245	212
5	293	196	142	228
Mean	309	172	225	211
*t* test, *p*	0.3	0.7	0.3	0.4

Paired *t* tests revealed no difference between movements to self-generated saccadic targets or explicit targets on any of these key kinematic measures

In summary, we have found a strong motor bias for eye movements to the pointer-only stimulus that it is in the same direction as the classical perceptual effect known as the Poggendorff effect. In addition, when a vertical inducing line is added to the pointer, both perceptual and motor responses are affected approximately equally by the Poggendorff illusion. These results agree with our findings using manual pointing and are consistent with the conclusion that there is a single underlying cause for the motor and perceptual effect.

## Experiment 2

Having found evidence suggestive of a relationship between motor and perceptual responses, we wished to further explore the way in which perceptual information can feed into motor planning. Therefore, in experiment 2, we varied the stimulus onset asynchrony (SOA) between presentation of the pointer-plus-landing line and that of the inducing line. Participants were instructed to make their saccade as soon as the pointer-plus-landing line appeared, so the inducing line could appear at various times during the saccade planning process or during the saccade itself. Eight different SOAs (0, 50, 100, 200, 350, 500, 650 and 800 ms) were randomly interleaved over trials. Since the trial terminated at 900 ms, the overall time the inducer was present covaried inversely with SOA. The following experiment measured motor response only, and the reported bias is the vertical distance between the true extrapolated position on the landing line and the participants’ point of regard following the initial saccade, measured in screen pixels (one pixel = 0.36 mm).

### Results

**Fig. 3:**
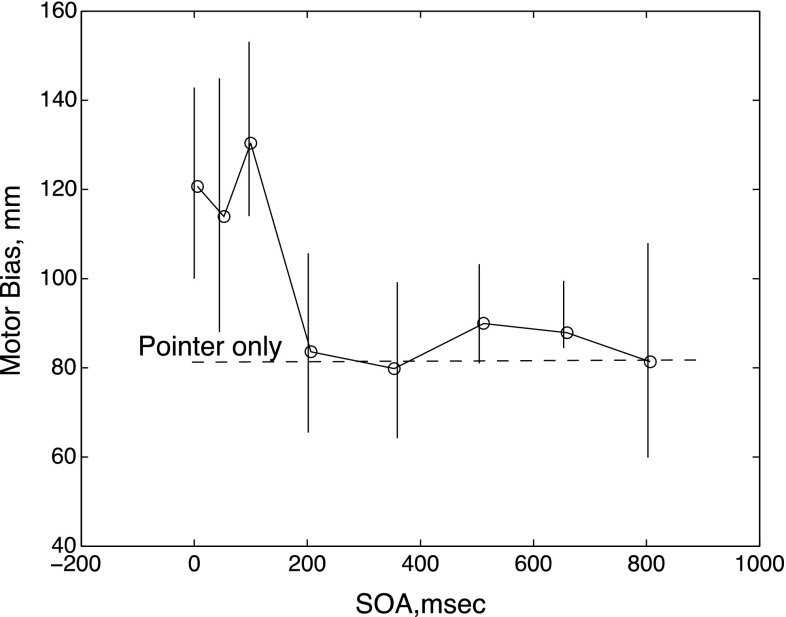
Results, averaged across the four participants from experiment 2, in which onset of the inducer was delayed after the appearance of the pointer and landing line. *Bars* indicate the standard error of the mean across participants. The *dashed line* shows performance in the pointer-only condition

Figure 3 shows average biases for eye movements with increasing onset asynchrony of the inducer. The points are averages over participants (*n* = 4) and trials (*n* = 10). All onset delays up to and including 100 ms produced similar strong Poggendorff effects upon the participants’ eye movements, but there was an abrupt decrease in this effect at a delay of 200 ms, at which point the impact of the inducer became effectively nil, with biases indistinguishable from the ‘pointer-only’ condition. Since the average latency of saccades was ~250 ms, these data suggest that the introduction of the inducer influenced only the initial phase of the saccade planning process, in agreement with the normal assumption that saccades are essentially ballistic and are not influenced by stimuli presented immediately before or after they have been launched. Our finding that a stimulus presented in close temporal proximity to the saccade target can influence the saccade trajectory agrees with experiments on the remote distractor effect (RDE; Walker et al. 1997), and in particular, the robust RDE found when the distractor is delayed by 50 ms and its disappearance with delays greater than 100 ms (Buonocore and McIntosh 2008).

## General discussion

We are not the first to find that saccadic eye movements can show biases similar to those of perception. Findlay and Hotopf (1985) asked participants to transfer their point of regard from the tip of a pointer to the termination of a horizontal target line oriented at either 45° or 135° with respect to the pointer (i.e., similar to Fig. 1d). A well-established perceptual bias (Obenai 1931) is that the pointer actually aligned with the tip of the 45° target line seems to point toward the ‘center of gravity’ (COG) of the target line. Findlay and Hotopf found that this was also true of the horizontal component of the eye movement. It is of particular interest that the effect was much smaller for a 135° pointer than one at 45°. In the latter case, a displacement of the horizontal saccade endpoint in the direction of the COG is the same as a saccadic undershoot; in the 135° case, the COG and undershoot effects are in opposition. It seems likely, therefore, that there are two effects in the Findlay and Hotopf experiment: One is a displacement toward the COG, and the other is a general undershoot when the target position is uncertain. This is exactly what we find in our experiment. There is a general undershoot, seen in the pointer-only condition similar to that of Findlay and Hotopf, and an additional Poggendorff effect when an inducing line is also present.

Previous work (Morgan 1999) has shown that inducing lines make no contribution to the Poggendorff effect when their intersection with the pointer is made invisible or bent. This finding suggests that a highly localized, orientation-selective mechanism must make a significant contribution to the Poggendorff effect.

The most surprising aspect of our results is that saccades to extrapolated targets were similar in their latency, maximum velocity and subsequent duration to those made to an explicit target. This argues that a perceptual process is involved in both cases and that the computation of a perceptual extrapolation takes no extra time.

Our findings add to the already considerable literature on illusions and action, but they do not fit neatly into any existing classification scheme. Bruno et al. (2008), reviewing studies of Muller-Lyer illusions, conclude that several factors determine whether manual pointing to the ends of the Muller-Lyer figure is, or is not, affected by the perceptual illusion. One factor is whether the stimulus is actually present when the response is performed or whether it is absent so that the response is memory driven. The latter favors the illusion. Our task is clearly visually driven, because the stimulus was present before, during and after the saccade. A memory-driven version would be one in which the stimulus was presented and then removed except for the initial fixation point and landing line for a variable delay period before the saccade was triggered by the removal of the initial fixation point. This procedure is practically guaranteed to produce a perceptually driven bias in the saccade, because the subject would have to select and remember a point on the landing line to use as a subsequent target. This was not the case in either of our experiments. Moreover, the results of the second experiment (Fig. 3) suggest that the presence of the inducing stimulus during the initial period (up to 100 ms) of saccade planning was crucial. Thus, our finding of a Poggendorff effect on saccades does not support the idea that memory is necessary for a motor illusion. On the other hand, it could reasonably be said that our stimulus requires the subject to form a perceptual representation to guide the response, since the target is virtual rather than explicit. That is, an ‘imaginary’ versus a ‘real’ target may be more important for the illusion than the involvement of memory per se.

A second factor mentioned by Bruno et al. (2008) is whether the starting position of the action is on the figure itself, or at some point outside the figure, the latter tending to weaken the effect of the context on pointing. Bruno et al. suggest that starting the action from outside the figure encourages an egocentric frame of reference, which reduces the illusory effect on action, consistent with mainly dorsal stream involvement (Goodale and Milner 1992). It is not entirely clear how this distinction maps on to our task, but a good case could be made for the starting point being within the figure, thus favoring the illusion. It can also be said that the extrapolation task is quintessentially one that cannot be performed in a strictly egocentric framework, since the target for the saccade is defined with respect to the angle of the pointer, not with respect to the body. Thus, the final programming of the saccade in an egocentric framework must be preceded by an allocentric representation of the stimulus configuration, and it is in the earlier stage that the perceptual bias exerts its effect. We have not yet performed an experiment where the initial fixation point was entirely outside the figure and the angle of the saccade thus different from that of the pointer. Once again, however, it is difficult to see how such a task could fail to show the Poggendorff bias, since the target for the saccade must be perceptually constructed.

Finally, Bruno et al. (2008) are dismissive of the idea (Franz 2001; Franz et al. 2001) that the illusion is decreased in closed-loop conditions (where the hand is visible as it moves to the target), because this factor has been confounded with memory- versus stimulus-driven paradigms. Our findings are consistent with the view that open-loop conditions favor an effect of context on action: first, because saccades are thought to be essentially ballistic, and second, because there is no feedback to tell the subject whether they have reached the ‘correct target’ or not, since the target is never made explicit. Indeed, this latter also applies to pointing responses, even with a visible hand, to the virtual (‘imaginary’) target of the Poggendorff illusion and may, thus, explain why these manual actions were also subject to the conventional bias in our previous study (Melmoth et al. 2009).

A further classification of illusions into those that do and do not affect motor behavior has been suggested (Dyde and Milner 2002; Milner and Dyde 2003), based on the finding that the simultaneous ‘tilt illusion’ affects the action of posting a letter through an aperture in the illusorily titled central stimulus. The suggestion is that illusory biases arising from processing such a cross-orientational inhibition in early visual areas may be inherited by the mechanisms for visuo-motor behavior in the dorsal stream, without having to go through ventral stream ‘perceptual’ processing. This could explain why the Poggendorff effect is found in saccades, since cross-orientational inhibition related to the misangulation of the pointer and inducing lines is one of the several factors that have been implicated in the missense of visual direction contributing to the perceptual Poggendorff effect (Morgan 1999).
